# Phylogenetic Analysis of C Type Lectin from *Toxocara* canis Infective Larvae and Comparison with the C Type Lectin Family in the Immune System of Mouse and Human

**Published:** 2018

**Authors:** Fazeleh ETEBAR, Seyed Hossein HOSSEINI, Fatemeh JALOUSIAN, Mohammad Mehdi RANJBAR

**Affiliations:** 1. Dept. of Parasitology, Faculty of Veterinary Medicine, University of Tehran, Tehran, Iran; 2. Dept. of Immunology, Razi Vaccine and Serum Research Institute, Karaj, Iran

**Keywords:** Nematoda, *Toxocara canis*, C type lectin, Phylogenetic analysis

## Abstract

**Background:**

C type lectin (CTL) family is a type of calcium-dependent proteins found in vertebrates and invertebrates. The objective of this study was to perform a comparative analysis and phylogenetic inferring for understanding the similarities and differences of carbohydrate recognition domain (CRD) domain of *Toxocara canis* CTL and other nematodes, and similar C type lectin involved in the immune system of mouse and human as their host.

**Methods:**

The female *T. canis* was retrieved from the 2–6 months puppies (Department of Parasitology, Faculty of Veterinary Medicine, University of Tehran, 2015). To collect *T. canis* eggs, the worms were cultured for 5 d until they were embryonated. The hatching process was accelerated for collecting the stage 2 larvae, and the larvae were cultured for a week. A cDNA library was made from the total mRNA of *T. canis* infective larvae. The PCR amplification for C type lectin gene was performed and the amino acids were analyzed using the alignment method and the construction of phylogenetic tree.

**Results:**

The suspension sample maintained at 30 ºC for four weeks could embryonate 90%–100% of eggs. *T. canis* CTL gene was 657 bp in length and encoded a protein with 219 amino acids. The CTL of species of Strongylida order were closely placed in the tree, whereas the members of Ascaridida orders were located in a separate branch. High levels of similarity (36%–44%) and conservation of C type lectin from *T. canis* with mouse and human C type lectins. Its C type lectin showed a higher similarity with asialoglycoprotein receptor (ASGPR), macrophage lectin, dendritic cell-specific intercellular adhesion molecule 3-grabbing nonintegrin (DC-SIGN), MINCLE receptor of mouse and human.

**Conclusion:**

Analysis of CRD domain of C type lectin protein could make a better understanding of their role in the interaction of nematode parasite with their hosts.

## Introduction

Toxocariasis is a cosmopolitan disease caused by Ascarid nematodes named *T. canis* and *T. cati*, which are gastrointestinal parasites of canids and felids, respectively. Embryonated ova infect paratenic hosts including human and mice, but the larvae do not mature and migrate through a soft tissue and then reside as an arrested larvae for a long time extend to years (1). In the tissue of the paratenic hosts, the larvae shed proteins, including a family of glycoproteins containing C type lectin domain, from their cuticle (2). This family has been found among both parasitic and free-living nematodes, as well as other invertebrate and vertebrate animals.

C type lectin is a family of calcium-dependent glycoproteins that their carbohydrate recognition domain (CRD) binds to mono and oligosaccharides. They recognize pathogens and involve in the cellular interactions through protein-carbohydrate interactions (3). Proteins from this family contain one or more CRD with 110–130 amino acid residues, the existence of multiple CRDs exceed the avidity of proteins to their ligands (4). Although these proteins share structural homology, they have variable amino acid sequences within the CRD. The diversity of CRD can improve the interaction and binding of lectin with different carbohydrates (5). This domain has a characteristic double-loop connected by two highly conserved disulfide bonds, which have up to four Ca^++^ binding sites. The proteins with C type lectin domains in mammalian as nematode hosts are a family associated with immune responses.

This family can be categorized by their functional and structural characteristics into five main classes. Class I contains Lecticans interacting with carbohydrate and protein ligands through a hyaluronan binding domain and a C type lectin domain. Four lecticans have been identified including aggrecan, versican, neurocan, and brevican that all four glycoproteins are expressed in the nervous system (6). Class II named collections consists of C type lectin and collagen domains. This subfamily contains proteins such as Lung surfactant proteins A and D (SP-A and SP-D), Mannan-binding lectin (MBL) and conglutinin involved in innate immune defense through binding pathogens carbohydrates and then aggregation, and phagocytosis (7, 8). Class III or selectins (CD62), are a subfamily of cell adhesion glycoproteins involved in the inflammation processes. There are three types of selectins including L-selectin (leucocytes), E-selectin (endothelial cells), and P-selectin (platelets). Class IV contains CD23 that is an antibody receptor found on IL4 activated B cells, activated macrophages, and eosinophils and have a role in IgE regulation (9). Class V consists C type lectins which act as receptor-mediated endocytosis, and include the asialoglycoprotein receptor (ASGPR), the macrophage mannose receptor, DC-SIGN (dendritic cell-specific intercellular adhesion molecule 3-grabbing nonintegrin), and the macrophage galactose-binding lectin (MGL) in myeloid cells (5).

Recently, C type lectins have been identified from parasitic worms. The function of this family involves in various biological activities, including impede host immune system by interfere host-parasite interaction (10) and other functions such as binding to host ligands (11), antibacterial function (12), and interfere with the mucin that secreted in response to the worm infection in gastrointestinal nematode infections (13). The parasite lectins from *Ancylostoma ceylanicum* play a role in reproduction (14). C type lectin is one of the secreted glycoproteins of the *T.* canis larvae (L2) with 219 amino acids.

We aimed to perform a comparative analysis and phylogenetic inferring for understanding the similarities and differences of CRD domain of *T. canis* CTL and other nematodes, *Loa loa* XP003142450, *Brugia malayi* XP001892052, *Trichinella spiralis* XP003380979, *Trichuris suis* KFD52311, *Strongyloides ratti* CEF60330, *Dictyocaulus viviparus* KJH47372, *Ancylostoma ceylanicum* AAD51335, *Necator americanus* AAY58318, Laxus *oneistus* AAX22004, *Caenorhabditis elegans* NP507547, *Caenorhabditis brenneri* EGT51156, *Ancylostoma duodenale* KIH65040, *Nippostrongylus brasiliensis* ACS37723, *Heligmosomoides polygyrus* ACS37721, *Oesophagostomum dentatum* KHJ91188, *Haemonchus contortus* CDJ85736, *Caenorhabditis remanei* XP003095271, *Caenorhabditis briggsae* XP002648922, *Ascaris suum* ADM49197, *T. canis* ctl 4 AAD31000, *T. canis* ctl 1 AAB96779, and also similar C type lectin involved in the immune system of mouse and human as their host.

## Materials and Methods

### In vitro culture of adult *T. canis*

The study was approved by *Ethics Committee of* faculty of Veterinary Medicine, University of Tehran.

The female *T. canis* was retrieved from the 2–6 months puppies following the method described by Ponce Macotela (15). Worms were isolated and then were washed with paintbrush and normal saline for three times in order to remove the debris and contamination from their surfaces. Twenty female worms were separated and transferred to 20 ml of preheated sterile RPMI 1640 (Gibco) (at the Department of Parasitology of Faculty of Veterinary Medicine, University of Tehran, 2015), the media contained penicillin (100 U/ml), streptomycin (100 μg/ml), amphotericin B (50 μg/ml) (Sigma-Aldrich Chemie GmbH, Germany). The adult female *T. canis* was maintained in tissue culture flasks (Sigma - Aldrich canted neck flasks, 25 cm^2^) in an incubator at 37 ºC and 5% carbon dioxide. To collect *T. canis* eggs, the worms were transferred to a new culture flask every 24 h for 5 d. The collected medium was centrifuged, then the collected eggs were washed twice with 1.0% formaldehyde, and incubated in PBS (pH: 7.2), a solution containing 1.0% formaldehyde and amphotericin B (50 μg/ml) until eggs were embryonated.

To determine the effects of different temperatures and media, eggs were divided into three batches. The groups differed in temperature. The three groups with 1% formalin were maintained at 30 °C, 27 °C, and 23 °C at 60% humidity. An inverted microscope monitored samples until the development of eggs. We counted the numbers of embryonated eggs at the end of sixth week.

### In vitro culture of infective larvae

In order to induce egg hatching, the eggs were washed three times with sterile distilled water. A suspension containing 1000 eggs was mixed with sodium hypochlorite (4%–14% free chlorine) and maintained at 37 °C in an incubator while the mixture was shaking. After 20 min the outer layer of eggs was removed. Then the eggs were washed with sterile distilled water until the chlorine was unnoticeable. Then for accelerating the hatching process, the remained coated layer of eggs were mechanically destroyed using a hand-held homogenizer, then the mixture of larvae and debris was added to the Baermann apparatus for separating the motile larvae.

The collected larvae were cultured at 37 °C incubator with 5% CO_2_ in the medium RPMI 1640 supplemented with 1% (w/v) glucose, 100 U/ml penicillin, 100 μgr/ml streptomycin, and 50 μgr/ml amphotericin B for a week (16).

After a week, for collecting the stage 2 larvae, the media was centrifuged at 200 gr for 10 min at 4 °C, and then the larvae were maintained in −80 °C until it was used for mRNA extraction.

### cDNA synthesis and C type lectin PCR amplification

The mRNA was extracted from 1000 infective larvae by following the manufacturer’s instructions (Tripure Isolation Reagent, Roche). A cDNA library was made from the mRNA of *T*. *canis* infective larvae. First, the PCR amplification was performed for cytochrome oxidase 1 gene to confirm that the cDNA made belongs to the *T*. *canis.* Then the PCR amplification for C type lectin gene was performed in a 25 μl volume containing 1 μl cDNA sample and 23 μl reaction mixture, containing two micromols of each primer (forward) 5′,<GGATCCATGATGATCGCCGC>-3′ and (reverse) 5′-, <GAATTCTTAGAGAGGTCTCT> -3′. The PCR conditions were as follows: an initial denaturing step (95 °C for 5 min) followed by 35 cycles, with each cycle consisting of denaturation at 94 °C for 45 sec, annealing at 67 °C for 45 sec, elongation at 72 °C for 45 sec, and a final extension at 72 °C for 10 min. For the detection of the PCR amplicons, 8 μl of the PCR products were separated using 1% agarose gel electrophoresis. The PCR products were purified using the quick PCR products purification kit (MBST, Iran).

The C type lectin template of *T. canis* was digested with *BamHI* and *EcoRI*, and consequently, the TA plasmid vector was double digested with the same restriction endonucleases. The PCR product of the C type lectin gene was cloned to TA plasmid using the rapid DNA ligation kit (Fermentase, Lithuania).

### Sequence alignment and Phylogenetic analysis

The sequence chromatograms were analyzed using the Chromas software version 3.1 and compared to those registered in the GenBank, using the Basic Local Alignment Search Tool (BLAST).

The C type lectin domain sequences contain 110–130 amino acids of *T. canis*, and other nematodes were obtained from GenBank (https://www.ncbi.nlm.nih.gov/). The sequences were aligned using Tcoffee with Clustal W2 method, then analyzed by the CLC sequence viewer software version 6.6.2. The conserved and variable regions were evaluated by CLC software. Phylogenetic tree was constructed by MEGA 6 software package using the neighbor-joining (NJ) algorithm, and bootstrap was set on 1000 (17).

## Results

### In vitro culture of adult *T. canis* and infective larvae

*T. canis* adult worms were cultured for 5 d. There were significant differences between the numbers of the embryonated eggs at different temperatures (*P*<0.0%). In average, 90%–100% of the eggs in samples kept at 30 °C were embryonated, whereas for the second and third group cultured at 27 °C and 23 °C, only 40%–50% and 10%–20% of eggs were embryonated, respectively. Increasing in temperature caused a rise in the number of developed eggs. The suspension eggs mixed with 1% formalin and amphotericin B (50 μg/ml) at 30 °C could be fully embryonated within 6 wk without fungal or bacterial contaminations.

### Electrophoresis and sequencing of CTL from Toxocara canis infective larvae

**Fig. 1:**
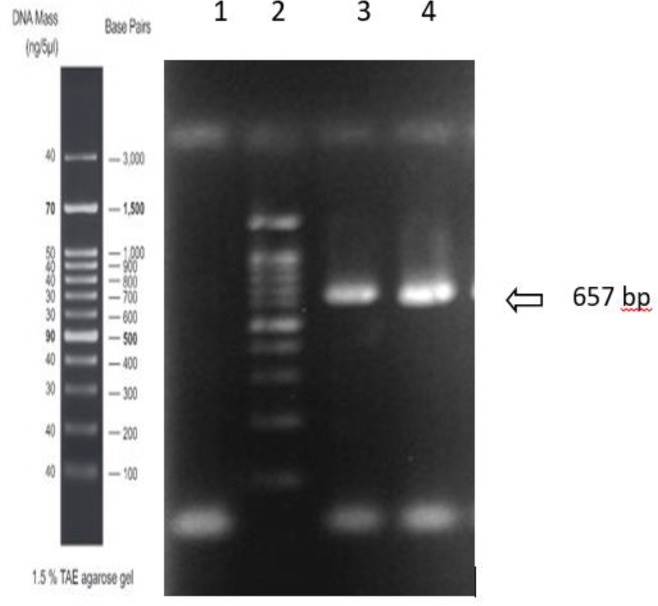
Gel electrophoresis of a PCR product 657 bp segment of C type lectin gene of *T. canis*. From left to right: lane 1 negative control, lane 2 100-bp DNA marker, lanes 3 and 4 PCR product 657 bp segment of C type lectin gene.

A cDNA library was derived from *T. canis* larvae (L2). The CTL sequence was submitted to the GenBank with the accession number of KU852582 (Fig. 1). *T. canis* CTL gene was 657 bp in length and encoded a protein with 219 amino acids with a molecular weight of about 24 kDa. The CTL sequence includes a signal peptide of 18 residues, and a CTL domain starting at residue 81 to 216. The sequencing result of C type lectin retrieved from the isolates of *T. canis* from dogs in Iran I showed few differences in amino acid sequences at position 22 and 147 with the excretory-secretory C-type lectin TES-32, isolated from United Kingdom (AAB96779) and differed in amino acid at position 56 with proteoglycan core protein, isolated from Japan (BAA31253).

### Phylogenetic Analysis

The topology of tree shows four groups. In group 1, the close relationships among *L. loa*, *T. spiralis*, *B. malayi*, *T. suis*, *S. ratti, A. ceylanicum*, *N. americanus*, L. oneistus in C type lectin sequences, although the sequences in *L. loa*, *B. malayi*, *T. spiralis* are more similar to each other than the other members of this group.

**Fig. 2:**
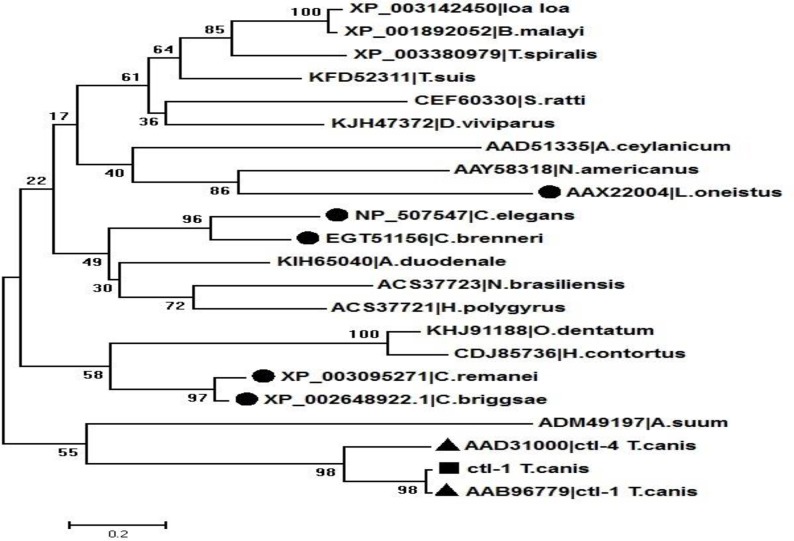
Phylogenetic tree of CTLs of parasitic and free-living nematodes. Phylogenetic tree of nematode C type lectin was constructed using the neighbor-joining (NJ) algorithm (with a bootstrap value of 1000 replicates) based on differences in the C type lectin amino acid sequences of parasitic and free-living nematodes. Species names and accession numbers place at the tips of the branches. The CTLs of free-living nematodes are marked by ●, CTLs of *T. canis* that retrieved from the GenBank are shown ▲ and the CTL sequenced in our study is shown by ■. :.

The nearest relative to the mentioned cluster is group 2 that includes *C. elegans, A. duodenale*, *N. brasiliensis*, *H. polygyrus*. *O. dentatum, H. contortus*, *C. remanei, C. briggsae*, showing less relationship to other nematodes located in a separate branch. Forth cluster separated as a distinct branch includes *A. suum*, CTL 1, CTL 4, *T. canis* and C type lectin sequenced in our study. The CTL 1 sequence and C type lectin sequenced in our study located in sister branch and the most similar sequences were CTL 4 and C type lectin of *A. suum* (Fig. 2).

### Similarities with mammalian C type lectins

The *T. canis* C type lectin sequences were used to search for proteins with C type lectin domain that involved in the immune response of mouse and human as the paratenic hosts of *T. canis*. The sequences were aligned using Tcoffee with Clustal W2 method, then analyzed using CLC sequence viewer software version 6.6.2. The conservation scale in this result was defined as a score from 0% to 100%.

The closest similarity to *T. canis* C type lectin was for macrophage lectin, asialoglycoprotein receptor, CD23, galactose and N-acetylgalactosamine-specific lectin, macrophage C type lectin, Mincle receptor, mannose-binding protein and DC-SIGN. The sequences retrieved from the GenBank and BLAST results showed 21%-31% similarity to *T. canis* C type lectin sequence, although if similarity between sequences is expressed based on percent positive substitutions, they would show a similarity of 36%–44% (18).

## Discussion

Lectins are molecules extremely diverse in amino acid sequences. Their functions in the processes of carbohydrate recognition are influenced by their versatility. *T. canis* CTL contains a single CRD, whereas the CTLs of some nematodes such as *A. ceylanicum*, *N. americanus*, *H. contortus*, *O. dentatum*, *C. elegans* have two or more CRDs. *T. canis* C type lectin has been considered as an important protein in immunogenicity in host-parasite interactions (11).

The Phylogenetic results from C type lectin of nematodes revealed that the high polymorphism in this gene could relate to differences in their *taxonomic positions as well as* their life cycles. The most nematode C type lectin sequences registered in the GenBank databases belong to Strongylida order (*A. ceylanicum* AAD51335, *N. americanus* AAY58318, *A. duodenale* KIH65040, *O. dentatum* KHJ91188, *H. contortus* CDJ85736, *N. brasiliensis* ACS37723, *H. polygyrus* ACS37721, *D.viviparus* KJH47372). The Phylogenetic relationships based on CRD domain among different nematodes showed that the gastrointestinal nematode species of Strongylida order were closely placed in the tree, although the other member of this order*, D. viviparus* as a nematode living in lung were in near branches with nematodes belongs to Filarioidea (*loa loa* XP003142450, *B. malayi* XP001892052) and Trichocephalida (*T. suis* KFD52311, *T. spiralis* XP003380979). *Loa loa* and *B. malayi* as filarial nematodes were clustered with each other in a clad and *T. spiralis* as parasite that lives within a muscle cell was more similar to the clad mentioned. The Members of Ascaridida orders were located in a separate branch. The Phylogenetic analysis of identified parasitic nematode CTLs showed that the similarities within the species of a family are more common in comparison with distinct families.

These proteins involved in innate defense to encounter the pathogens of their environment (19). In contrast, the proteomic analysis of parasitic nematodes revealed that they have fewer proteins with C type lectin domain modified according to their life cycles, to play a role in parasite-host interface (20, 21).

**Fig. 3:**
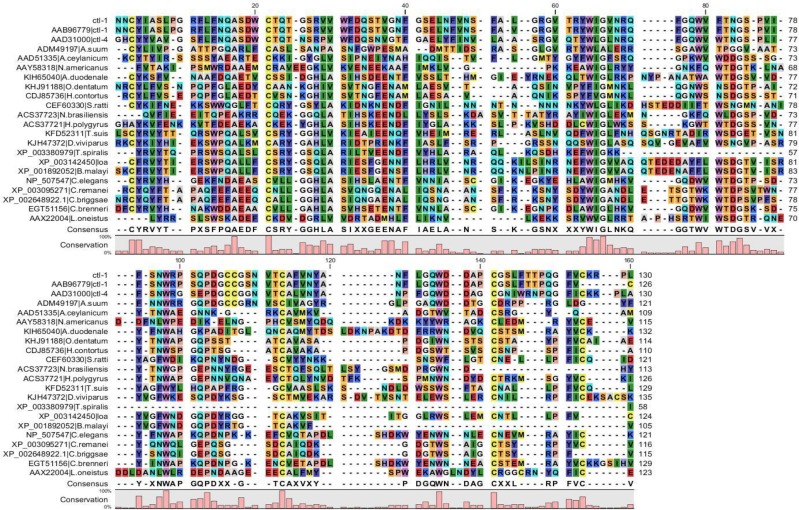
Aligned amino acid sequences of *T. canis*, C type lectin, and C-type lectin of other nematodes. Conserved cysteine residues are highlighted in yellow. The carbohydrate recognition motif as conserved residues that bind to sugar is indicated by an A bracket. The conservation score is indicated at the bar graph for each column of the alignment

**Fig. 4:**
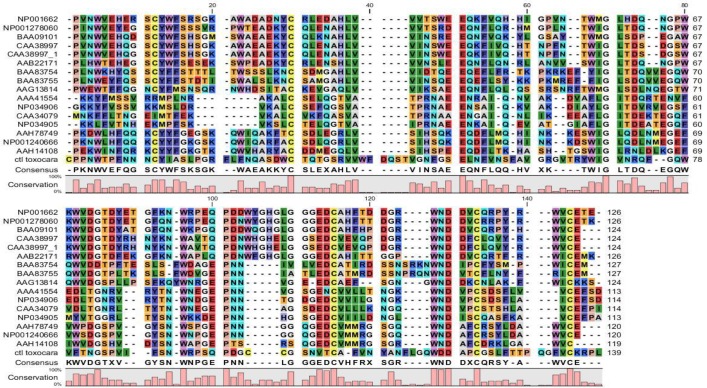
Aligned amino acid sequences of *T. canis* C type lectin and C type lectin proteins of immune system. Conserved cysteine residues are highlighted in yellow. The carbohydrate recognition motif as conserved residues that bind to sugar is indicated by an A bracket. The conservation score is indicated at the bar graph for each column of the alignment

For further alignment analysis, the sequences of the *T. canis* CTL and the proteins with C type lectin domain involved in mammalian immune responses were aligned. These results showed a high conservation at four cysteine residues forming two disulfide bonds and these residues are considered critical for the fundamental structure. Calcium binding site two is the most important residues for binding sugar. The amino acids that have a carboxylic acid group as the side chain, consist of **aspartic acid** and **glutamic acid,** are often involved in the Ca^++^ binding site in the CRD. The conserved motifs that bind to sugar are EPN and its substitutes QPD and WND are important motifs in the calcium binding reaction (22). These motifs determine the monosaccharide binding specificity of C type lectins, EPN binds Man/GlcNAc and QPD binds Glc/Gal. *T. canis* CTL has QPD motif similar to ASGPR and macrophage C type lectin, and others have EPN. *T. canis* CTL showed a higher similarity with ASGPR, macrophage lectin, DCSIGN, MINCLE and less similarity with MBP, although *T. canis* CTL is considered to bind both mannose and GalNAc monosaccharides (10), also *T. canis* CTL showed about 28% similarity with lecticans including bravican, aggrican, neurocan and versican specifically expressed in the nervous system (Fig. 3, 4).

It is essential for *T. canis* as a nematode live for a long time in the tissues such as nervous tissues, to escape from the host immune system, and CTL is one of the most glycoprotein secreted by this nematode in larval stages. Likewise, Free-living nematodes encode abundant proteins with C type lectin domain. Nematode CTLs are considered to play several functions such as tissue recognition during their migration in host tissues involving in evading from immune system and by their similarity to the host proteins such as selectins. They can interfere cell adhesion, which is important for leukocyte recruitment to the sites of inflammation and injury. Some inconsistent existence in the grouping of sequences may become resolve by identifying more C type lectin sequences within different order of nematodes.

## Conclusion

The analysis of CRD domain of C type lectin protein of *T. canis* could expand our understanding of host-parasite interaction and immune responses in toxocariasis. It is important for *T. canis* larvae to escape from the host immune system and CTL has been considered as an important protein in immunogenicity of *T. canis*. CTL of *T. canis* shares similar sequence with proteins of immune system of their hosts such as mouse and human. A deep insight into phylogenetic relationships and study the sequence variability and similarity are crucial for study of sequence evolution and identification of functional regions of this protein.
